# Characteristics of Lake Chad Level Variability and Links to ENSO, Precipitation, and River Discharge

**DOI:** 10.1155/2014/145893

**Published:** 2014-11-27

**Authors:** Churchill Okonkwo, Belay Demoz, Sium Gebremariam

**Affiliations:** Beltsville Center for Climate System Observation (BCCSO), Atmospheric Science Program, Howard University, Washington, DC 20059, USA

## Abstract

This study used trend, correlation, and wavelet analysis to characterize Lake Chad (LC) level fluctuations, river discharge, El Niño Southern Oscillation (ENSO), and precipitation regimes and their interrelationships. Linear correlation results indicate a negative association between ENSO and LC level, river discharge and precipitation. Trend analysis shows increasing precipitation in the Lake Chad Basin (LCB) but decreasing LC level. The mode of interannual variability in LC level, rainfall, and ENSO analyzed using wavelet analysis is dominated by 3-4-year periods. Results show that variability in ENSO could explain only 31% and 13% of variations in LC level at Kindjeria and precipitation in the northern LCB, respectively. The wavelet transform coherency (WTC) between LC level of the southern pool at Kalom and ENSO is statistically significant at the 95% confidence level and phase-locked, implying a cause-and-effect association. These strong coherencies coincide with the La Niña years with the exception of 1997-1998 El Niño events. The WTC shows strong covariance between increasing precipitation and LC level in the northern pool at a 2- to 4-year band and 3- to 4-year band localized from 1996 to 2010. Implications for water resource planning and management are discussed.

## 1. Introduction

The Lake Chad Basin (LCB) (latitude 6°N–24°N; longitude 7°E–24°E) is part of the Sahel, a semiarid region that is prone to drought [1]. Lake Chad (LC), a closed lake at the center of the LCB, is highly sensitive to hydroclimatic events [2]. The LCB hydroclimatic system is influenced by many factors including precipitation, river discharge, climate indices, teleconnection, and anthropogenic factors [3]. However, formal treatment of anthropogenic contributions to the shrinking of LC is treated elsewhere [4]. Many studies have shown that precipitation in the Sahel region of Africa is influenced by oceanic conditions impact on atmospheric circulations especially Atlantic Multidecadal Oscillation AMO [5] and El Niño Southern Oscillation ENSO [6]. This study will focus on the temporal variability of precipitation as reflected in time series of precipitation and river discharge in the LCB and global El Niño Southern Oscillation (ENSO) events.

According to the International Panel on Climate Change (IPCC) Fourth Assessment Report, decreases in the size of lakes can be attributed primarily to human use and declining precipitation [7]. Isiorho et al. [8] reported that precipitation is one critical element that determines the amount of infiltration for groundwater recharge and runoff (river flow) to the phreatic aquifers southwest of LC. Also, the Komadugu and Yobe Rivers southwest of LC now flow for six months of the year instead of nine [9]. The frequent severe drought in the Sahel region, of which LC is a part, is modulated by high-frequency climate variability in ENSO [6].

The ENSO system, a coupled cycle of atmosphere and ocean [10], has been linked to global climatic anomalies [11]. This phenomenon has received huge attention due to its apparent catastrophic impacts including flooding and droughts. Studies on the role of ENSO events and the Pacific index in modulating precipitation in the Sahel region include those by Caminade et al. [6, 12]. El Niño results in the weakening of West African Monsoon (WAM) flow and creates a dry condition across the Sahel region. La Niña, on the other hand, creates a wet condition through the enhancement of Walker circulation [13]. Hwang et al. [14] studied the link between ENSO index and lake levels, documenting the response of three lakes—Hulun in northern China, Bosten in western China, and Ngangzi in eastern Tibet—to 1997-1998 El Niño events on interannual timescales. Eltahir [15] examined the impact of ENSO on the hydrology of lakes and the Nile Basin in East Africa. Also, Küçük et al. [16] reported a significant correlation between the North Atlantic Oscillations (NAO) index and Turkish lake levels using a wavelet approach.

Recently, the Sahel region has shown some signs of recovery from the droughts of the 1980s [17]. Some studies have attributed the recent greening in the Sahel region to increasing rainfall in the past decade, global warming [18], and sea surface temperature variability [17]. Nicholson [19] (2005) used a Tropical Rainfall Measuring Mission (TRMM) dataset to study the link between the recent greening of the Sahel and increasing rainfall. According to [19], the Sahara region remained relatively dry, unlike the western Sahel, which has exhibited a marked recovery. According to [20], Sahelian rainfall is characterized by interannual variations with fewer rain events within the 20-year dry period (1970–1989). Also, [21] reported resilience in the Sahelian ecosystem after the droughts of the 1970s and 1980s.

One of the necessary tasks in hydrology is the analysis of precipitation and river flow time series [22]. Time series of river discharge incorporate some important hydrological parameters such as precipitation, temperature, and changes in land cover [23]. There is a relationship between variability in water level of continental lakes and global climate changes [24].

Most previous studies on the changes in the size and level of LC are based on either hydrological models [4, 25] or imperial regression techniques [26]. While these studies have documented the historical changes in the size of LC, our understanding of the lake's hydrology is still poor [4]. Moreover, there is still a gap in the characterization of the relationship between ENSO and LC level. Wavelet analysis was used to fill this gap by identifying dominant scales of LC level variability that could lead to a better understanding of LCB hydrology. The nonstationary nature of rainfall variability in this region, ENSO, LC level and river inflow fluctuations make it advantageous to study LC using wavelet analysis. The most important advantage of wavelet analysis is that, unlike classical spectral analysis, which requires the restrictive assumption of stationarity, the wavelet approach focuses on time series that change over time [27].

This paper examines the connection between ENSO, rainfall, river discharge, and LC level. The hypothesis is that ENSO events have a significant effect on fluctuations in LC level. The scientific question addressed is whether there exists a statistically valid link between El Niño events, rainfall, river discharge, and Lake Chad level. The characterization of ENSO and its interrelationship with LC behavior could also assist water resource planning and management in the LCB. The rationale for characterizing LC level variability is to use the knowledge of the driving forces in planning for future climate change and sustainable water resource management.

The datasets, preparation, and study approach will be described in Section 2 of this paper.  Section 3 presents results of statistical analysis and the continuous wavelet transform and analysis.  Section 4 discusses the physical mechanism and Section 5 discusses the results and suggests possible applications.

## 2. Data and Methodology

### 2.1. Study Area and Data

**Figure 1:**
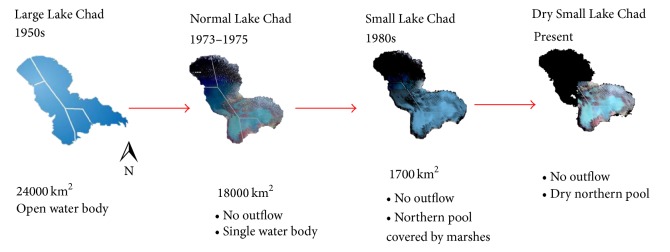
Schematics of the state of Lake Chad (modified from Landsat 5 images; courtesy of NASA).

A summary of the historical changes in LC is given in Figure 1 with the classification of various states of the lake as proposed by Singh et al. [4, 28]. The figure shows the dramatic decrease in lake size from about 24,000 km^2^ in the 1950s (Large Lake Chad) to about 18,000 km^2^ in the early 1970s (Normal Lake Chad). Drought during the late 1960s and early 1970s led to the splitting of the single lake into northern (Sahara-arid) and southern (Savanna-humid) pools around 1975 [4]. Recently, [4] added a new category called a Dry Small Lake Chad with the northern pool permanently dry most of the year.

**Figure 2:**
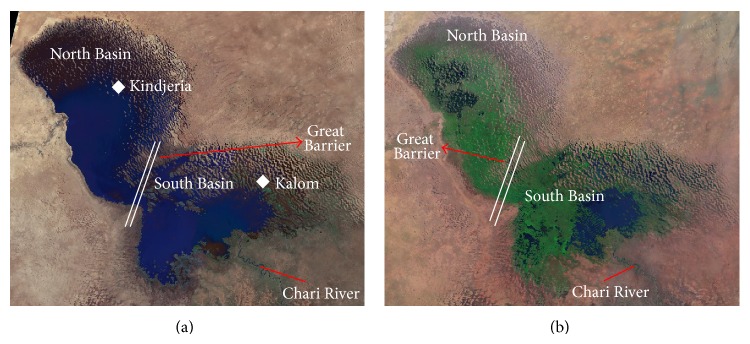
Landsat 5 images of Lake Chad: (a) January 1973 and (b) May 2003 (modified from Landsat 5 images; courtesy of NASA).

Studies of LC have documented its reduction in size 22,000 km^2^ to 300 km^2^ by the 1980s [29], the presence of the ridge referred to as the “Great Barrier” (Figure 2) by Olivry et al. [30] that runs between the southern and northern parts of the lake, the splitting of the lake into two smaller lakes when the inflow is below the barrier [25], and the inflow from the southern parts of the basin through the Chari River (Figure 2) that significantly determines the lake's behavior [25].

Recently, [4] used a combination of hydrological model, satellite, and field data to reconstruct the past levels of LC. The satellite measurement was from Topex/Poseidon satellite, a joint NASA/CNES (National Aeronautic and Space Administration/Centre National d'Etudes Spatials, France) altimetry mission launched in 1982. The northern and southern pools of LC water levels were based on reconstructed levels at Kalom and Kindjeria, respectively (Figure 2), and were digitized from this hydrological modeling study by Lemoalle et al. [4] to complement for years with no data from Topex/Poseidon satellite. The figures from [4] were scanned as a raster image that is then digitized using geographic information system (GIS). The digitization was carried out using point mode operation at maximum annual lake level. Postprocessing was applied to the digitized map by checking lake levels against the source figure for accuracy. The digitized data was used in the ground-water vulnerability. Long-term monthly gridded (0.5° × 0.5° latitude-longitude) precipitation datasets (version 3.01) produced by the Center for Climatic Research at the University of Delaware [31, 32] were also used in the study. The Multivariate ENSO Index (MEI) [33] was used to quantify the effect of ENSO events on precipitation and LC level. This is an index of several observed variables including sea surface temperatures, cloudiness, precipitation, winds, and sea surface pressures.

### 2.2. Methodology

**Figure 3:**
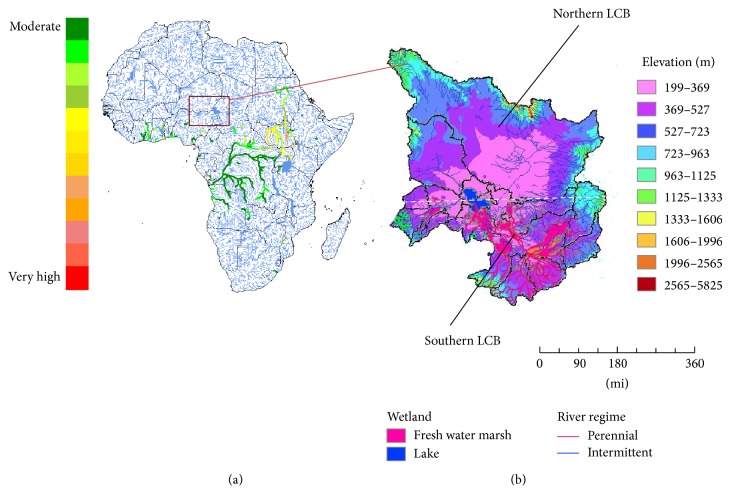
Study area. (a) River basin catchment map of Africa showing the Lake Chad Basin area (square), major rivers (blue), and runoff on October 12, 2012 (green-red); (b) LCB showing the major tributaries, the elevation, and the southern and northern parts of the lake (see text for details).

As part of the data preparation, area-weighted averaging was applied to the University of Delaware's gridded monthly time series. This has the advantage of minimizing the spatial data gaps in a semiarid region. Due to the split of LC into northern and southern pools, we divided the LCB into northern and southern basins (Figure 3) using the barrier described by Gao et al. [25]. This division enabled us to partition the highly variable precipitation and surface flow in the LCB [34] into the northern and southern basins, respectively.

Also, the physical terrain of the LCB with mountainous ranges at the borders means that precipitation ultimately recharges the lake through either surface flows by way of the tributaries or ground water recharge (Figure 3). Aggregated July, August, and September (JAS) annual rainfall totals in the northern and southern LCB were used. The relationship between LC level and the hydrologic variables was explored by conducting correlation, trend, and time series analysis. Using wavelet analysis (a multiscale nonstationary process), the interannual variability of precipitation, lake levels, Chari River discharge, and ENSO was examined by decomposing their time series into frequency space following the program developed by Torrence and Compo [35].

A summary of the basic theory of continuous wavelet transform (CWT), cross-wavelet transform (XWT), and wavelet transform coherency (WTC) following [35, 36] is given as

(1) $$\begin{array}{c} W\left(\tau ,s\right)=\frac{1}{{\left(s\right)}^{1/2}}\overset{+\infty }{\underset{-\infty }{\int }}X\left(t\right)\psi^{*}\left(\frac{t-\tau }{s}\right)dt, \end{array}$$

where *ψ*(*t*) is the mother wavelet defined by *τ*, the transition parameter corresponding to the position of the wavelet and *s*, the scale dilation parameter that determines the width of the wavelet. The variability of the dominant mode over time was determined using the Morlet wavelet with a wave number *w*_0_ = 6 as the mother wavelet. The choice of Morlet wavelet is based on its localization in time and frequency, making it a good tool in extracting features [36].

For two time series (*M*_*x*_ and *N*_*y*_), the XWT is given as

(2) $$\begin{array}{c} W^{MN}=W^{M}W^{N*}, \end{array}$$

where (^*^) stands for the complex conjugate. The XWT helps in determining whether the two time series are statistically significant by the Pearson correlation coefficient. WTC is the cross correlation between two time series and indicates how coherent the XWT is in time-frequency space [36]; it is given by Torrence [37] as

(3) $$\begin{array}{c} R_{n}^{2}\left(s\right)=\frac{{\left|\left(s^{-1}W_{x}^{MN}\left(s\right)\right)\right|}^{2}}{S\left(s^{-1}{\left|W_{x}^{M}\left(s\right)\right|}^{2}\right)\cdot S\left(s^{-1}{\left|W_{x}^{N}\left(s\right)\right|}^{2}\right)}, \end{array}$$

where the smoothing operator *S* is written as

(4) $$\begin{array}{c} S\left(W\right)=S_{\text{scale}}\left[S_{\text{time}}\left(W\left(s\right)\right)\right]. \end{array}$$

Smoothing along the wavelet axis is given by *S*_scale_ while smoothing along the time axis is given as *S*_time_. The design of the wavelet smoothing operator is given by Torrence [37] as

(5) $$\begin{array}{c} {\left.S_{\text{time}}\left(W\right)\right|}_{s}={\left.\left(W_{n}\left(s\right)*c_{1}^{-t^{2}/2s^{2}}\right)\right|}_{s},\\ {\left.S_{\text{scale}}\left(W\right)\right|}_{t}={\left.\left(W\left(s\right)*c_{2}\prod \left(0.6s\right)\right)\right|}_{n}, \end{array}$$

where ∏ is the rectangle function; *c*_1_ and *c*_2_ are normalization constants, while 0.6 is the empirically determined scale decorrelation length for the Morlet wavelet [35]. The major difference between XWT and CWT is that cross-wavelet power measures the common power while coherency is a measure of intensity of covariance between two time series.

## 3. Results

### 3.1. Time Series, Trend, and Correlation Analysis of ENSO, Lake Level, and Precipitation

**Figure 4:**
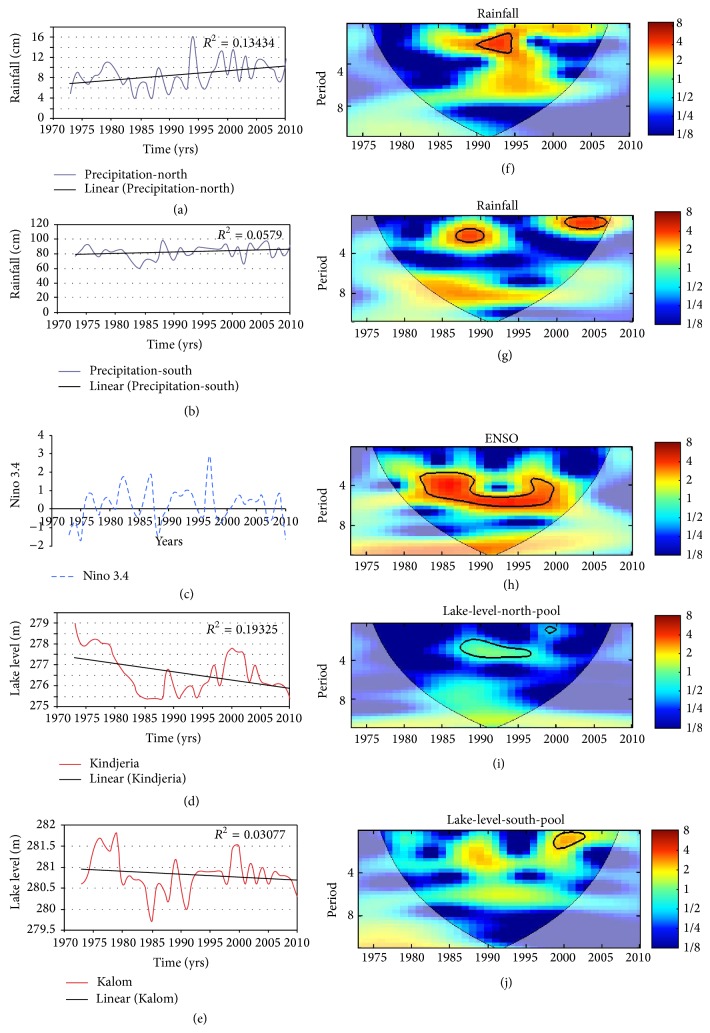
Standardized time series (left) of (a) rainfall in the northern LCB, (b) rainfall in the southern LCB, (c) ENSO, (d) LC level at Kindjeria, and (e) LCL at Kalom and their respective continuous wavelet transforms (f), (g), (h), (i), and (j) (right). The thick contour enclosed regions are greater than 95% confidence for a red-noise process. The thin solid line indicates the “cone of influence,” where edge effects become important.

The time series and CWT of precipitation, lake levels, and ENSO are shown in Figure 4. The JAS rainfall in the northern LCB shows large variability, with less than 4 cm in 1984, corresponding to one of the severe droughts that have plagued the Sahel region (Figure 4(a)). In the southern LCB, the impact of the severe drought of the early 1980s was especially pronounced in 1984 (Figure 4(b)). The alternation in ENSO events between the El Niño (warm) and La Niña (cool) phases is evident in Figure 4(c). There was a continuous decrease in LC level from 1979 to 1985 in the northern pool (Figure 4(d)). In the southern pool, the decrease in the lake's level from 1979 to 1985 is less dramatic (Figure 4(e)); here, the drop in water level was due in part to decreased inflow from the Chari River, the main tributary to LC [38].

Also, the time series of rainfall in the North Basin (Figure 4(a)) and South Basin (Figure 4(b)) display common variability from 1972 to 2010 with the exception of the spike in rainfall in the North Basin in 1993. There appears to be some recovery in rainfall beginning in 1999 in the northern LCB (Figure 4(a)) and around 1991 in the southern LCB (Figure 4(b)), corresponding to the onset of recovery in Sahel rainfall as reported by Nicholson [13]. The minimum rainfall in 1984 coincides with minimum lake levels in the North and South pools of LC. The reported breakup of LC into northern and southern pools around 1975 [4] appears to have resulted from the late 1960s Sahel drought.

The precipitation trend shows an increase in the northern and southern parts of the LCB, with weak coefficients of determination (*r* ^2^) of 0.13 and 0.05, respectively. The time series fluctuate above and below the trend line in all cases. A fitting of linear trend lines shows a decreasing lake level trend at Kindjeria and Kalom with coefficients of determination (*r* ^2^) of 0.19 and 0.03, respectively, for the period 1973–2010. The decreasing trend for Kindjeria, though weak, is still statistically significant at the 90% confidence level. The LC level at Kindjeria is thus decreasing even with the increasing precipitation trend in the northern LCB. It is thus essential to understand the driving forces of this decreasing lake level and its impact on the surrounding ecosystem in order to plan for future climate change and sustainable water resource management in the LC region.

The trend and visual comparisons shown in Figure 4 give a sense of the time series of the ENSO and hydrological variables. Quantitative analytical techniques based on correlation were used to assess the relationships between ENSO, LC level variability, rainfall, and Chari River discharge. From the results, lake level at Kalom (*r* ^2^ = 0.31) and lake level at Kindjeria (*r* ^2^ = 0.39) both had a weak but statistically significant relationship with Chari River discharge (Table 1). This result shows that the Chari River can account for only 31% of LC level variability at Kalom, while precipitation accounts for 9%.

An apparent physical disconnect between LC level variability and precipitation can be seen in Figure 4. For example, a closer look at the LC level from 2005 at Kindjera (Figure 4(d)) shows decreasing lake level even with increasing precipitation (Figure 4(a)) in the North Basin. In contrast, Figure 4(e) shows that, instead of decreasing trend, the LC at Kalom has been slightly increasing along with the increases of precipitation in South Basin (Figure 4(b)) at the same period. An exception to this increasing trend can be seen around 2008/9, with increasing precipitation but decrease in lake level. These disconnects between rainfall and LC level can be explained by two factors. One is that even when Lake Chad is a closed basin, it is still linked to groundwater [8, 39]. As such, the lake level is significantly affected by anthropogenic pressure through groundwater extraction and diversion for irrigation purposes. The anomaly in LC level in Kindjera (north pool) on the other hand can be attributed to low lake level in the south pool well below the barrier that there is no inflow from north pool to south pool. This figure clearly indicates the existence of a turning point around 1984 with opposite trends before and after the point. There are, however, some signs of recovery at least in the south pool from around 1998/99 corresponding to onset of recovery in Sahel rainfall as reported by Nicholson [13].

There are strong correlations between Chari River discharge and precipitation in southern parts of the LCB (*r* ^2^ = 0.76), between lake level at Kalom and lake level at Kindjeria (*r* ^2^ = 0.66), and between precipitation in the northern part of the LCB and precipitation in southern parts of the LCB (*r* ^2^ = 0.65). The low correlation between precipitation in southern parts of the LCB and lake level at Kindjeria (*r* ^2^ = 0.05) and that between precipitation in northern parts of the LCB and lake level at Kalom (*r* ^2^ = 0.10) could be explained by the absence of a connecting physical hydrological mechanism.

Also, Leblanc et al. (2011) [2] had reported water transfer from the southern pool to the northern pool across the Great Barrier only when the south pool reached 280 m. Figure 4(e) shows that lake level at Kalom (southern pool) was less than 280 m from around 1980 to early 1990s. The decreased transfer of water can partly explain the low correlation between precipitation in northern LCB and lake level at Kindjeria (Table 1). The low correlation between lake level at Kindjeria and precipitation in southern LCB is also expected since LC level at Kindjeria largely depends on the over flow from the southern pool.

The correlations between the ENSO index and the LC hydrological variables in this study are negative and weak. However, correlations with rainfall south of the LCB (*r* ^2^ = −0.31) and lake level at Kindjeria (*r* ^2^ = −0.31) are both statistically significant at the 95% confidence level. Also, 14% (*r* ^2^ = −0.14) and 12% (*r* ^2^ = −0.12) of the fluctuations (decrease) in lake level at Kalom and Chari River discharge, respectively, are explained by ENSO variability. The results demonstrate that variability in precipitation south of the LCB, lake level at Kindjeria, Chari River discharge, and lake level at Kalom is significantly accounted for by ENSO variability.

The next step is to expand these time series into frequency space by applying CWT as a band filter to the time series. This will help in finding localized periodicities and in feature extraction. Figures 4(f), 4(g), 4(h), 4(i), and 4(j) show the CWT of rainfall in the northern LCB, rainfall in the southern LCB, ENSO, LC level at Kindjeria, and LCL at Kalom, respectively. The CWT showed common periodicity dominated by intra-annual and annual fluctuations mainly in the 1-year, 2-year, and 3- to 4-year bands. In addition, the peaks are significantly important with reference to the late 1980s drought in the region. These data suggest causality between these hydroclimatic variables ENSO, rainfall, river discharge, and LC level. The correlations and coherencies of these variables will be examined in subsequent sections. CWT will determine if these associations are merely coincidences by obtaining the frequency component of the hydroclimatic variability as a function of time. Wavelet coherency between two CWTs will address the statistical significance of the coherences and will also provide a confidence level against noise.

### 3.2. LC Level and Rainfall Variability

**Figure 5:**
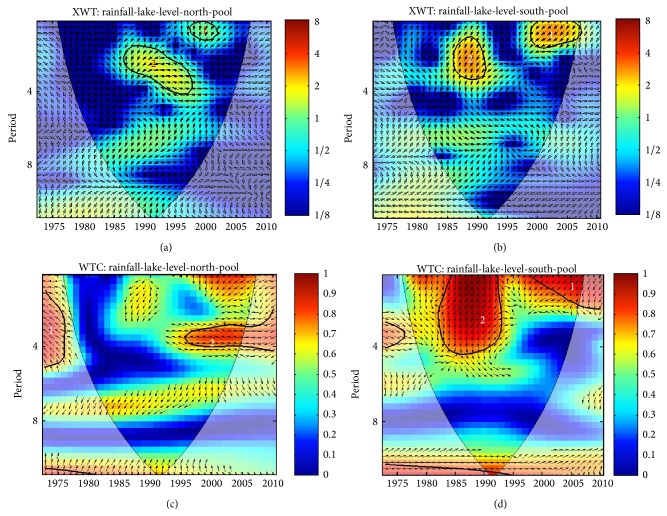
Precipitation and lake level cross-wavelet spectra: (a) northern LCB; (b) southern LCB and wavelet coherence; (c) northern LCB; and (d) southern LCB (in-phase pointing right, anti-phase pointing left, leading by 90° pointing straight down). The thick contour enclosed regions are greater than 95% confidence for a red-noise process. The thin solid line indicates the “cone of influence,” where edge effects become important.

The XWT and WTC between LC level and rainfall in northern and southern LCB are shown in Figure 5. According to [35], this is a representation of cross-correlation between the variables as a function of time and frequency. The phase difference between the lake level and precipitation is represented by the vectors, whereas the locally significant power of the red noise spectrum at the significance level of *α* = 0.1 is shown by the bold solid contour line [35]. The lighter black contour line is the cone of influence (COI) where edge effects are not negligible. The coherence power between two series is shown in the color code of red to blue (strong to weak).

The correlation analysis through XWT (Figure 5(a)) shows that LC levels were correlated highly with rainfall on the interannual scale at two bands: a 1-year band localized at 1997 and a 2- to 4-year band localized around the years 1985 to 1997. The localization for the 2- to 4-year band for the Southern Basin is around 1985–1990. This localization in the northern basin may be attributed to the strong ENSO event of 1983–1987, which led to intense drought in the Sahel region. According to [36], cause-effect relationships in XWT are indicated by phase lock oscillation. Since LC level and rainfall are in antiphase in the 2–4-year band in the north pool, it is safe to conclude that rainfall mirrors lake level.

The WTC shows strong covariance between increasing precipitation and LC level in northern pool at two bands: a 2- to 4-year band that is outside the COI and a 3- to 4-year band localized from 1996 to 2010 (Figure 5(c)). For the southern pool, there are also two significant bands: a 1- to 2-year band localized from 1996 to 2010 and a 1- to 4-year band localized between 1991 and 1994 (Figure 5(d)).

This strong positive covariance between wavelet coefficients indicates a positive association between LC level and precipitation. Also notice that there is an extensive region that is significant (Figure 5(d)), making it very unlikely that the association is by chance. These covariances are important as they correspond to years of increasing precipitation and LC level.

### 3.3. LC Level and ENSO Events

**Figure 6:**
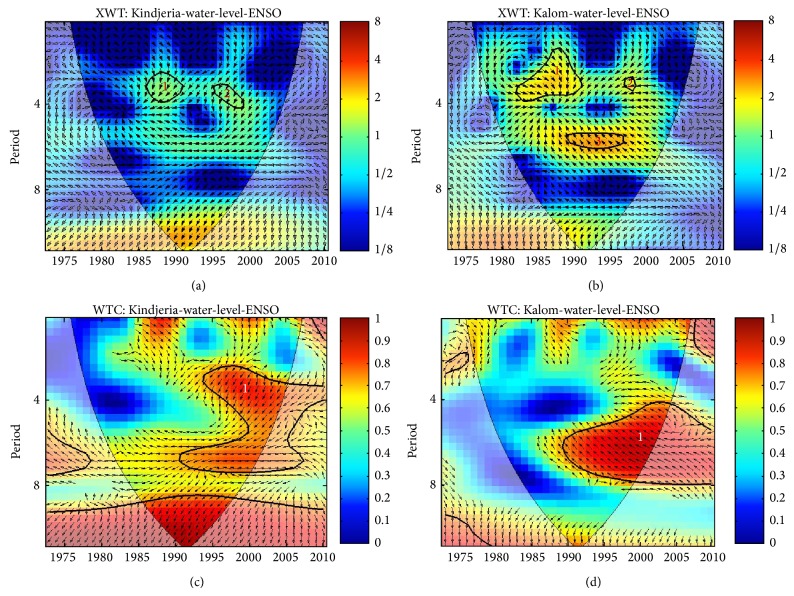
ENSO and lake level cross-wavelet spectra: (a) northern LCB; (b) southern LCB and wavelet coherence; (c) northern LCB; and (d) southern LCB. The thick contour enclosed regions are greater than 95% confidence for a red-noise process. The thin solid line indicates the “cone of influence,” where edge effects become important.

Cross-wavelet transform of ENSO and LC level in the North Basin shows high common power (good correlation) at two bands: a 3- to 4-year band localized from 1985 to 1987 and a 3- to 4-year band localized between 1995 and 1997 (Figure 6(a)). The XWT between LC level in the Southern Basin and ENSO (Figure 6(b)) shows three common powers that are statistically significant: a 2- to 4-year band localized between 1983 and 1987, a 3-year band localized around 1997, and a 6-year band localized between 1987 and 1997. The 6-year periodicity of LC level in the northern pool (Figure 6(a)) has less significant power but the phase is locked. As a result, a strong link between ENSO and LC level at Kindjeria is speculated. Also, ENSO spectral coherence is very strong in the 3- to 8-year band around 1990–2000 (Figure 6(c)). The region that is statistically significant in the WTC at the periodicity of 3 to 8 years (Figure 6(b)), however, suggests that the association is not by chance. The 2- to 4-year band and the 3-year band correspond to the last two strongest ENSO events, namely, 1983-1984 and 1997-1998.

For the south pool, ENSO spectral coherence is very strong in the 3- to 8-year band around 1990–2000 (Figure 6(d)). The association between lake level of the southern pool at Kalom and ENSO shows phase angles that are chaotic in the time periods of the 2- to 4-year band (Figure 6(c)). The 6-year band can be described as phase-locked with a strong region in the WTC that is statistically significant, also suggesting that the association is not by chance (Figure 6(d)). It should be noted that this strong coherence coincides with the La Niña years, with the exception of 1997-1998 El Niño events.

### 3.4. Precipitation and ENSO Events

**Figure 7:**
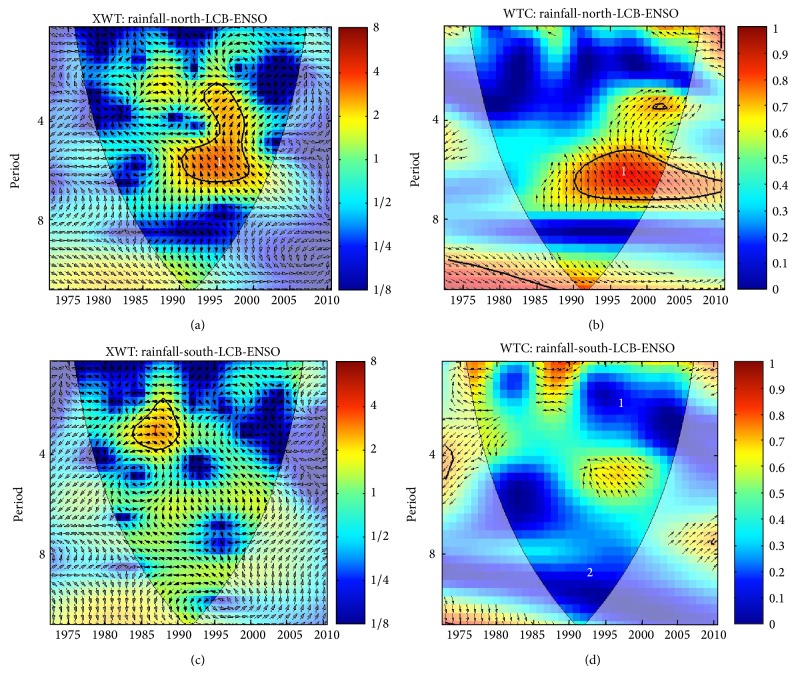
Precipitation and ENSO cross-wavelet spectra: (a) northern LCB; (b) southern LCB and wavelet coherence; (c) northern LCB; and (d) southern LCB. The thick contour enclosed regions are greater than 95% confidence for a red-noise process. The thin solid line indicates the “cone of influence,” where edge effects become important.

ENSO's role in modulating LCB precipitation in the North Basin (Figure 7(a)) shows high common power (good correlation) at one band, a 2- to 6-year band localized from 1990 to 1999. There is also one band (4- to 6-year) localized between the years 1985 and 1989 (Figure 7(c)) for the southern part of the LCB. ENSO spectral coherence with precipitation in the northern LCB is very strong in the 5- to 6-year band from 1990 to 2010 (Figure 7(b)), although 2000 to 2010 is outside the cone of influence. However, the coherency between precipitation and ENSO (Figure 7(d)) in the southern LCB is very weak. It shows two low bands of low frequency: a 2- to 4-year band localized between 1990 and 2010 and a 5- to 9-year band localized between 1980 and 2010. The phase relationship associated with signals in the 3- to 6-year band that are significant at the 95% confidence level shows an inconsistent phase angle (Figure 7(a)). The years 1990–1997 associated with signals in the 5- to 7-year period in the WTC (Figure 7(b)) have an extensive region that is significant at the 95% confidence level. This implies that the association between rainfall and ENSO in the northern LCB is not by chance. On the other hand, rainfall south of the LCB and ENSO is in antiphase in the 3- to 4-year band (Figure 7(c)), implying that ENSO mirrors rainfall. There is, however, no region that is significant at a 95% confidence level in the WTC.

The difference in coherency between the northern and southern LCB is noteworthy since it reflects the strong climate influence of ENSO events on the Sahel region (the northern LCB). This appears to be in agreement with [40], who documented ENSO influence on low-frequency rainfall variability in Sahel. According to [12], Sahel precipitation is linked to the developing and decaying phases of El Niño and La Niña, respectively.

### 3.5. River Flow and LC Level

**Figure 8:**
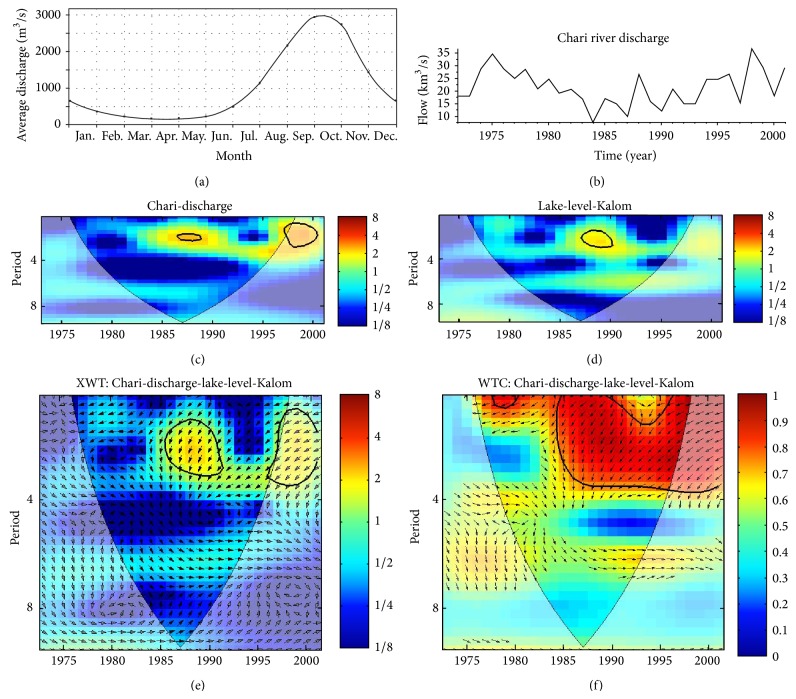
Chari River discharge: (a) mean monthly time series; (b) 1972–2000 time series; (c) CWT of Chari River discharge; (d) CWT of lake level at Kalom; (e) XWT; and (f) wavelet coherence. The thick contour enclosed regions are greater than 95% confidence for a red-noise process. The thin solid line indicates the “cone of influence,” where edge effects become important.

Figure 8 shows the discharge characteristics and wavelet results of the Chari River, the main tributary flowing into LC. Note the strong seasonality of the discharge with a peak around September-October that lags peak precipitation in the region (August) by about a month (Figure 8(a)). This is close to the results observed by Rodell et al. [41], who found a five-week lag between LC level and water storage in the highlands of the Chari-Logone drainage basin. According to a World Bank report [42], this decreased flow can be attributed to increased abstraction for human consumption. Notice the slight lag between the variability in LC level at Kalom (Figure 4(e)) and Chari River discharge (Figure 8(b)), which can be attributed to the time needed for the runoff to discharge to LC.

Figures 8(c) and 8(d) show the CWT of Chari River discharge and LC level at Kalom. The CWT showed common periodicity of intra-annual fluctuations in the 2-year band localized at 1987. The XWT correlation of LC level at Kalom with Chari River discharge shows high common power (good correlation) at two bands: a 3- to 4-year band localized between 1986 and 1990 and a 2- to 4-year band localized between 1995 and 2000 (Figure 8(e)). The spectral coherence is very strong in the 1- to 4-year band around 1985–2000. This strong coherency between Chari river discharge and LC level (Figure 8(f)) also shows phase angles that are pointing downward and sometimes chaotic. Again, as in previous sections, the XWT of the Chari River discharge in the 3- to 4-year band localized between 1986 and 1990 is noteworthy since this corresponds to the strong 1983–1987 ENSO events. It also corresponds to the year of lowest discharge of the Chari River in this study. The 2- to 4-year XWT band localized from 1995 to 2000 is a reflection of the intensity of covariance between increasing river discharge (Figure 8(a)) and LC level in the southern pool (Figure 6(d)). This suggests a phase of significant recovery from the record low levels in the late 1970s to early 1980s.

## 4. Physical Mechanism

In general, the results show that, during a positive ENSO event, July-August-September (JAS) rainfall over the southern and northern LCB declines. A similar result over East Africa shows decline in rainfall from October to December during the El Niño phase of ENSO [43]. The covariance between Chari River discharge and lake level in this study (*r* ^2^ = 0.78) is consistent with the reports by Hwang et al. [14, 44] on the role of river discharge and precipitation in modulating lake level. The correlation between ENSO and LC level during El Niño events from the wavelet analysis is in agreement with [24], who reported that variability in the water level of continental lakes could be affected by global climate changes.

Several studies have examined the teleconnection between rainfall variability in Sahel and variation in global sea surface temperature (SST) [45]. While it has been accepted that SST patterns play a significant role in rainfall variability in West Africa [46], there is still a debate regarding the major drivers [47]. In this study, the mode of interannual variability in LC level, rainfall, and ENSO analyzed using wavelet analysis is dominated by 3-4-year periods. For example, the detection of the 3-4-year band localized from 1985 to 1987 in the association between ENSO and LC level has not been documented previously. This is an interesting result because the correlation at a 3-4-year period occurs after the 1980s, when the variance in ENSO is strongest [37]. The physical mechanism responsible for this link is not yet clear. However, the Tropical Easterly Jet (TEJ) has been reported to be weaker in the El Niño years [48], which translates to more tropospheric convergence and less tropospheric divergence [49].

These conditions are unfavorable to convective precipitation systems in the region [50] and could explain the antiphase relationship between ENSO and rainfall in southern LCB (Figure 7(c)). This antiphase relationship indicates a cause-and-effect association. These results thus support earlier reports regarding the role of ENSO in modulating Sahel rainfall [51]. This link between LC level, precipitation, and ENSO at this low frequency adds to the understanding of LCB hydrology.

## 5. Discussion and Conclusions

We summarize our results as follows:

1. linear correlation results from this study indicate a negative association between ENSO and LC level, river discharge, and precipitation;
2. trend analysis shows increasing precipitation in the Lake Chad Basin (LCB) but decreasing LC level;
3. the strong 1997-1998 El Niño event decreased inflows downstream from the Chari River while La Niña years (2000–2004) saw increased inflow. This is partly a result of decreased precipitation in the El Niño years and increased precipitation in the La Niña years, respectively.

What should be done about the potential risks associated with the effect of ENSO on water resources in the LCB is the role of policy makers and not scientists. Yet scientists can help policy makers to evaluate what “critical” drought entails by laying out the element of risk. By characterizing the association between ENSO, precipitation, and Chari River, this study is proposing ENSO forecasting as a potentially powerful tool for water resource planning in the LCB. Substantial capacity can be developed by relevant governmental and nongovernmental agencies to recognize and announce ENSO forecasts to local farmers in time for proper planning. Researchers, forecasters, and policy makers actively engaged in finding water resource management solutions for the LCB face the challenge of ensuring effective implementation of an early drought warning mechanism in the LCB.

Also, the proposed interbasin water transfer from the Ubangui River Basin to the LCB [4, 25] can benefit from this characterization in a number of ways. First, the association between LC level, precipitation, and ENSO will enable managers to plan for the total water volume that can be released and retained based on ENSO forecasts. Regulation of river discharge across the interbasin boundary based on water needs of the receiving basin (the LCB) constitutes a nonstationary time series. Over time, wavelet analysis of this irregular but regulated time series of released water in association with other hydroclimatic factors could be a potential decision-making tool. The results from this study have thus provided an important benchmark for future studies.

Finally, while emphasizing the importance of ENSO in remotely influencing LC level variability, it should be pointed out that a much deeper understanding of the effect of other oceanic conditions like Atlantic Multidecadal Oscillation (AMO), Indian Ocean, and Mediterranean at different time-scales on Sahel precipitation is needed for a complete picture. However, the recognition of the negative response of LC level to El Niño event established in this study will be beneficial to forecasters and help the LC region adequately prepare for future dry period.

## Figures and Tables

**Table 1:** Correlations between rainfall (in northern and southern parts of the LCB), Chari River discharge, lake level (at Kalom and Kindjeria), and ENSO in matrix form (∗ and + denote significance at 99.5% and 95% confidence levels, respectively).

Variable	ENSO	Precipitation in LCB (south)	Lake level at Kalom	Chari River Discharge	Precipitation in LCB (*north*)	Lake level at Kindjeria
ENSO	1	−0.31^+^	−0.14	**−0.12**	−0.13	−0.31^+^
Precipitation in LCB (South)	−0.31^+^	1	0.09	0.76^∗^	0.65^∗^	0.05
Lake level at Kalom	−0.14	0.09	1	0.31^∗^	0.10	0.66^∗^
Chari River discharge	**−0.12**	0.76^∗^	0.31^+^	1	0.58^∗^	0.39^∗^
Precipitation in LCB (North)	−0.13	0.65^∗^	0.10	0.58^∗^	1	−0.00
Lake level at Kindjeria	−0.31^+^	0.05	0.66^∗^	0.39^∗^	**−0.00**	1
